# Paraganglioma of the urinary bladder: Case report and literature review

**DOI:** 10.1002/iju5.12185

**Published:** 2020-07-04

**Authors:** Hirofumi Kurose, Kosuke Ueda, Mami Uegaki, Naoyuki Ogasawara, Hisaji Kumagae, Katsuaki Chikui, Makoto Nakiri, Kiyoaki Nishihara, Mitsunori Matsuo, Shigetaka Suekane, Jun Akiba, Hirohisa Yano, Tsukasa Igawa

**Affiliations:** ^1^ Department of Urology Kurume University School of Medicine Kurume Japan; ^2^ Department of Pathology Kurume University School of Medicine Kurume Japan

## Abstract

**Introduction:**

Paraganglioma of the urinary bladder is a very rare disease accounting for 0.06% of all bladder tumors. Owing to their rarity and symptomatic variability, preoperative diagnosis is often difficult.

**Case presentation:**

A 70‐year‐old male was referred to our department for hematuria. Cystoscopy showed a non‐papillary broad‐based tumor. Computed tomography and magnetic resonance imaging revealed a 32‐mm bladder tumor at the top of the bladder, which suggested muscle‐invasive bladder tumor. We diagnosed muscle‐invasive bladder cancer or urachal carcinoma, and transurethral resection of the bladder tumor was performed. At the initiation of transurethral resection of the bladder tumor, the systolic blood pressure was elevated to over 200 mmHg. The pathological findings revealed paraganglioma of the urinary bladder, and afterward, a partial cystectomy was performed.

**Conclusion:**

We herein reported the case of paraganglioma in the bladder whose blood pressure became extremely elevated during transurethral resection of the bladder tumor. In addition, we analyzed important factors for preoperative diagnosis using 162 cases reported in Japan.

**Abbreviations & Acronyms**
CI: confidence interval
CT: computed tomography
GAPP: grading of adrenal pheochromocytoma and paraganglioma
HR: hazard ratio
MRI: magnetic resonance imaging
PUB: paraganglioma of the urinary bladder
TURBT: transurethral resection of the bladder tumor
WI: weighted imaging

**Keynote message**

Young age, the number of symptoms, and micturition attacks were identified as independent predictors for preoperative diagnosis of paraganglioma in the bladder.

## Introduction

Paraganglioma is a rare neuroendocrine tumor and is reported to account for 18% of pheochromocytomas, with 10% of the cases occurring in the bladder.^1^, ^2^ PUBs account for approximately 0.06% of bladder tumors.^3^, ^4^ Over 250 cases have been reported in Japan. In some cases of PUBs, serum catecholamine levels are normal, and the patients are asymptomatic; therefore, preoperative diagnosis is often difficult.

Here, we report a case in which abnormally high blood pressure was caused during a TURBT, and a diagnosis of PUBs was determined after surgery. In addition, of 262 cases reported in Japan, 162 cases for which detailed information was collected were analyzed to study the important factors for preoperative diagnosis.

## Case presentation

**Fig. 1:**
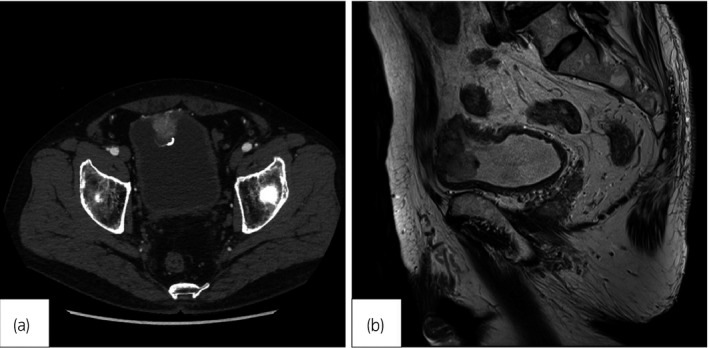
(a) CT revealed a 32 × 30‐mm bladder tumor covered with calcification at the top of the bladder. (b) MRI revealed a low intensity signal on T2‐WI, which suggested muscle‐invasive bladder tumor.

A 70‐year‐old male was referred to our department for hematuria. He had a history of hypertension for which he had received an oral antihypertensive agent. There were no episodes of micturition attacks. Cystoscopy showed a non‐papillary submucosal tumor covered with calcification on the posterior wall. Urinary cytology showed a class II lesion. CT revealed a 32 × 30‐mm bladder tumor at the dome of the bladder, and hyperdensity in the early phase and wash out in the late phase were observed (Fig. 1). MRI revealed a low intensity signal on T1‐WI and T2‐WI, which suggested muscle‐invasive bladder cancer (Fig. 1). Based on these findings, we diagnosed muscle‐invasive bladder cancer or urachal carcinoma, and TURBT was performed. His usual blood pressure was approximately 120/90 mmHg. At the initiation of TURBT, his systolic blood pressure was elevated to over 200 mmHg, and the tumor was easily bleeding.

**Fig. 2:**
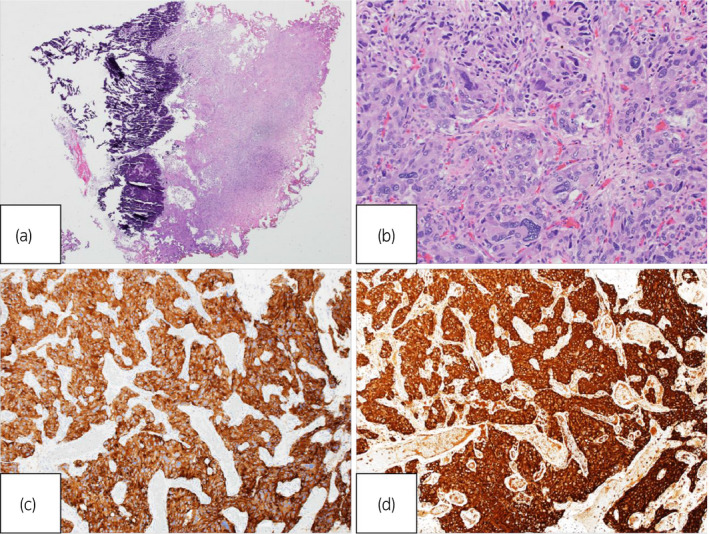
Microscopic findings reveal typical histological appearance of PUBs (Hematoxylin and eosin stain: (a) ×40, (b) ×200). An immunohistochemical study revealed positive findings for (c) synaptophysin, and (d) chromogranin A.

The pathological analysis revealed eosinophils that were associated with a fine vascular plexus and grew in a funicular pattern against partial necrotic tissue. The mitotic figure was inconspicuous (Fig. 2). An immunohistochemical study revealed positive findings for S‐100, chromogranin A, and synaptophysin and negative findings for CK7, p63, and GATA 3. The Ki‐67 labeling index was less than 1%. Based on these findings, PUB was diagnosed. The GAPP score was 5.

After TURBT, ^123^I‐IMBG scintigraphy showed no residual tumor, and the serum noradrenaline levels were slightly elevated at 682 pg/mL; however, both the serum and urine adrenaline showed normal values. Since the tumor invaded the muscle layer, a partial cystectomy was performed. There was no change in blood pressure during surgery. Although a histopathological analysis showed residual pheochromocytoma, the margin was negative. Postoperatively, the patient’s serum noradrenaline levels were normal.

## Discussion

Pheochromocytomas are rarely seen in extra‐adrenal locations, with 1% discovered in the urinary bladder.^5^ Owing to their rarity and symptomatic variability, PUBs are often misdiagnosed. In the present study, we analyzed important factors for preoperative diagnosis using 162 cases reported in Japan. The patient characteristics of these 162 cases are shown in Table 1.

**Table 1:** Patient characteristics and association between preoperative diagnosis and clinical factors

Parameter	Overall	Preoperative diagnosis: paraganglioma, *n* (%)	Preoperative diagnosis: not paraganglioma, *n* (%)	*P*‐value
Patients, *n*	162	63 (38.9%)	99 (61.1%)	
Sex				0.197
Male	81 (50.0%)	27 (33.3%)	54 (66.7%)	
Female	81 (50.0%)	36 (44.4%)	45 (55.6%)	
Age at diagnosis [years], median (range)	52 (7–96)	48 (7–80)	59 (17–96)	<0.001
<52	78 (48.2%)	42 (53.9%)	36 (46.1%)	0.0002
>52	84 (51.8%)	21 (25.0%)	63 (75.0%)	
Symptoms				
Hypertension	63 (38.9%)	33 (52.4%)	30 (47.6%)	0.008
Gross hematuria	57 (35.2%)	13 (22.8%)	44 (77.2%)	0.99
Micturition attacks	51 (31.5%)	42 (82.4%)	9 (17.6%)	<0.001
Number of symptoms				<0.001
0	36 (22.2%)	30 (83.3%)	6 (16.7%)	
1	89 (54.9%)	55 (61.8%)	34 (38.2%)	
2	29 (17.9%)	15 (51.7%)	14 (48.3%)	
3	8 (4.9%)	8 (100%)	0 (0.0%)	
Max tumor diameter [mm], median (range)	25 (3–100)	38 (10–100)	22 (3–97)	0.0002
>25	88 (54.3%)	43 (48.9%)	45 (51.1%)	0.006

With regard to sex, previous studies have reported that the disease was more common in women, whereas there was no difference in the sex ratio in the cases reported in Japan.^6^, ^7^ In PUBs, hypertension, hematuria, and micturition attacks are three cardinal manifestations.^6^ However, the present analysis showed hypertension in 38.9% of cases, hematuria in 35.2%, and micturition attacks in 31.5%. There were only 4.9% of cases presenting all three manifestations, and 22.2% did not present any of the three manifestations. There has been no consensus on the most common site of occurrence, although Das *et al*. cited the dome and the trigone as sites of occurrence.^6^ The case reports in Japan showed the following locations for occurrence of a PUB: the posterior wall (30.1%), the dome (21.6%), the anterior wall (19.1%), the lateral wall (13.6%), the trigone (11.1%), and the neck (3.7%).

A preoperative diagnosis of PUB was made in 63 of the 162 (38.9%) cases. The useful factors for preoperative diagnosis were young age, hypertension, micturition attacks, the number of symptoms, and large tumor size. In particular, in the cases where micturition attack was noted, a preoperative diagnosis was made in over 80% of the cases. Neither hematuria nor the tumor site was a significant finding. Univariate and multivariate analyses for preoperative diagnosis of PUBs are shown in Table 2. Univariate analysis for preoperative diagnosis of PUBs revealed that micturition attacks alone was significant predictors for preoperative diagnosis of PUBs, but hypertension alone was not. Moreover, multivariate analysis demonstrated that young age, the number of symptoms, and micturition attacks alone were identified as independent predictors for preoperative diagnosis of PUBs. In the present case, in which preoperative hypertension and hematuria had been observed and the blood pressure had been controlled by an antihypertensive agent, paraganglioma was not included in the differential diagnosis based on the clinical symptoms; the patient was not diagnosed with paraganglioma until the results of intraoperative investigation and histopathological testing were analyzed.

**Table 2:** Univariate and multivariate analysis for preoperative diagnosis of PUBs

Parameter	Univariate		Multivariate	
	HR (95% CI)	*P*‐value	HR (95% CI)	*P*‐value
Sex, male	0.59 (0.31–1.10)	0.095		
Age at diagnosis, <52 [years]	3.49 (1.82–6.70)	0.0002	2.61 (1.17–5.84)	0.019
Number of symptoms		0.0015		0.0006
0	1	–	1	–
1	2.45 (1.03–5.80)	–	1.18 (0.45–3.08)	–
2 or 3	6.00 (2.15–16.7)	–	5.63 (1.95–16.2)	–
Symptom				
Only hypertension	1.49 (0.66–3.38)	0.337		
Only micturition attacks	18.5 (4.18–82.2)	<0.0001	23.8 (4.49–89.3)	<0.0001
Max tumor diameter, >25 [mm]	1.95 (1.03–3.68)	0.037	1.21 (0.54–2.73)	0.647
Site of tumor	1.42 (0.71–2.86)	0.315		

In the World Health Organization 2017 classification, all pheochromocytomas have been redefined as malignant tumors with the potential to metastasize. It has been reported that calcification is highly likely to be malignant, as in this case. PUBs are reported to have a relatively high rate of malignancy (15.5%).^6^ The present case report in Japan showed a similar result (14.8%). Recently, the usefulness of the GAPP score has been reported.^8^ The malignant potential of the present case was moderate according to the GAPP score, and continuous follow‐up of the patient was recommended.

Partial cystectomy is a general treatment method for PUBs. Since PUBs occur in the muscle layer, complete removal by TURBT alone would be considered difficult. In this case, the pathological examination after partial resection showed residual tumor, although ^123^I‐IMBG scintigraphy showed no residual tumor after TURBT. In the case of a functioning tumor, similar to the present case, changes in blood pressure during the TURBT procedure can be expected. PUB cases presenting all three of the cardinal manifestations are not common. Diagnosis preoperatively based on clinical symptoms, tumor size, and characteristics of the tumor is considered important.

In conclusion, we herein reported the case of paraganglioma in the bladder whose blood pressure became extremely elevated during TURBT, and analyzed significant clinical factors for preoperative diagnosis of paraganglioma in the bladder.

## Conflict of interest

The authors declare no conflict of interest.
